# First case of nickel-metal hydride battery ingestion in child

**DOI:** 10.1093/jscr/rjad079

**Published:** 2023-05-02

**Authors:** Martha Lok-Yung Hui, Justin Ho-Yin Ng

**Affiliations:** Mildura Base Public Hospital, Mildura, Australia; University of Edinburgh, Edinburgh, UK; Bendigo Health, Bendigo, Australia; University of Sydney, Camperdown, Australia

## Abstract

We describe a case of a 12-year-old female with a past history of autism spectrum disorder who presented to the emergency department of a rural hospital in Australia after ingesting two nickel-metal hydride (NiMH) batteries at home. Hitherto, no literature has described any gastrointestinal complications related to NiMH battery ingestion. This paper aims to provide insight into the management of NiMH battery ingestion and to increase the awareness of the need for prompt management to prevent further damage to the gastrointestinal tract.

## INTRODUCTION

Battery ingestion is very common among children and can cause serious complications or even death. The most common type of battery ingested is button batteries as they can be found in a number of electronic devices and toys. As such, nickel-metal hydride (NiMH) battery has gained increasing popularity because of its rechargeable property and high-energy density. Nonetheless, its implication on the onset and severity of erosive gastritis remains unknown given no documented literature up to date. We report a case of erosive gastritis secondary to NiMH battery ingestion in a child.

## CASE REPORT

**Figure 1:**
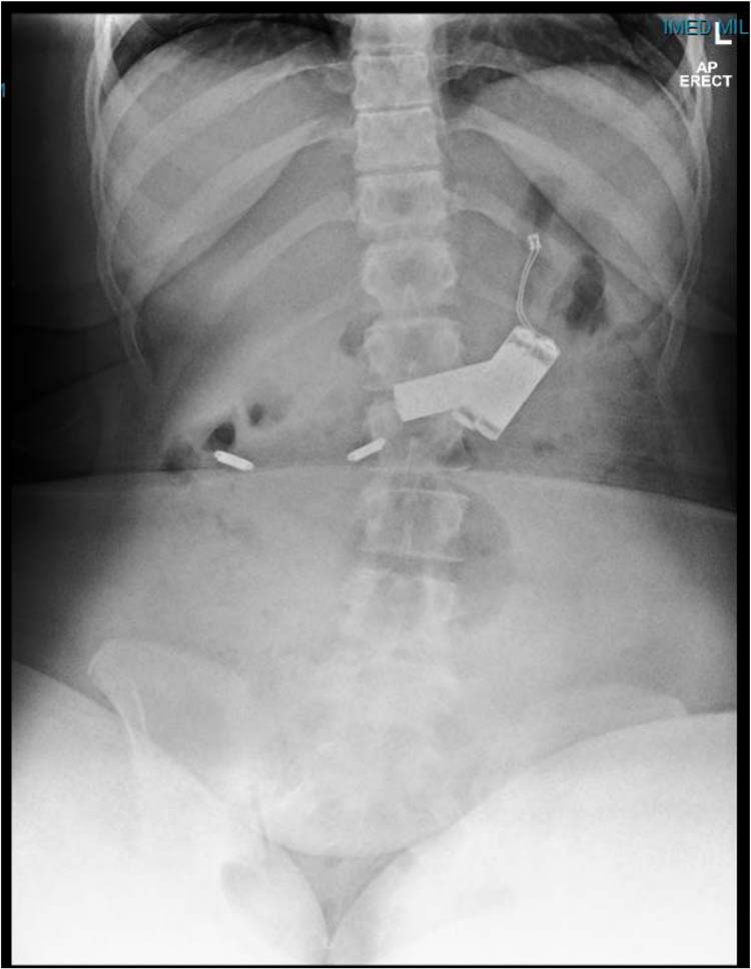
Abdominal X-ray demonstrating four battery-shaped foreign body at the level of L1/L2.

**Figure 2:**
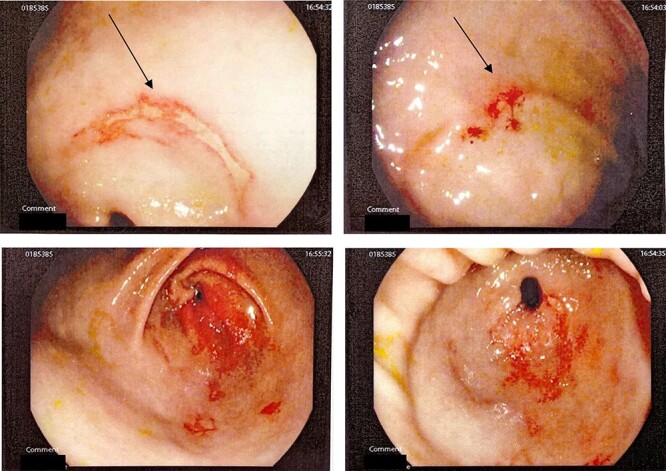
Gastroscopy showing extensive burns in antral and greater curvature of stomach.

This is a case of a 12-year-old Caucasian female with a background history of autism spectrum disorder presented to the emergency department of a rural hospital in Australia after the ingestion of two NiMH batteries. This is the third presentation of battery ingestion in 2 weeks. She was asymptomatic and did not complain of abdominal pain at the time of review. On examination, she had a soft abdomen with mild epigastric tenderness and guarding. Abdominal radiograph showed the presence of two battery structures projecting to the left of the upper abdominal area overlying L1/2 discs (Fig. 1). To the right of L2, there are two other round devices resembling metallic-type foreign bodies. A gastroscopy was performed 3 hours after her presentation. The gastroscopy revealed extensive superficial erosion to the antral and greater curvature (Fig. 2). Two NiMH batteries were retrieved. However, the other two smaller foreign bodies have passed the jejunum proximally and were unable to be retrieved. She was discharged home on sucralfate, pantoprazole and ColonLYTELY to promote passage of the remaining foreign bodies. Abdominal X-ray was repeated after 1 week, which showed no further foreign objects retained in the patient’s body. She recovered uneventfully with no development of any residual symptoms.

## DISCUSSION

Battery ingestion is common among children and is fatally dangerous, not only because of potential obstruction and chemical leaks in the GI tract but also the chemical interactions of the battery with bodily fluids. Cases of button battery ingestion are widely reported but there is none on NiMH battery. This is the first documented case of the ingestion of NiMH batteries. We aim to discuss the potential risk of NiMH batteries remaining in the digestive system.

NiMH batteries have a high metal content, in which it is composed of a cathode of nickel oxide hydroxide (NiOOH), an anode of hydrogen absorbing alloys (MH_2_), a potassium hydroxide (KOH) electrolyte and a high content of other rare earth elements such as lanthanum and cobalt [1]. The overall reaction is shown as follows:

MH_2_ + 2NiOOH ⇆ M + 2Ni(OH)_2_

Comparing to alkaline batteries, NiMH batteries can deliver a higher current with a smaller loss of battery capacity. Therefore, they are often used in high-current-drain devices such as mobile phones, digital cameras and computers [2, 3]. The concentration, pH of the alkaline agent and the duration of contact are the major determinators of the severity of the chemical burn in the digestive system. Nolan *et al*. [4] showed that battery leakage can happen as early as 2 hours after submersion in mercury batteries. Khalaf *et al*. [5] also suggested that the risk of gastric injury is 4.5 times higher among patients whose battery retention time is over 12 hours. Hence, surgical interventions are warranted to retrieve ingested batteries if spontaneous passage failed. The NiMH battery is considered to be safer than lithium-ion battery as various protection mechanisms are employed in its design [6]. However, the oxidation product nickel hydroxide is listed as a carcinogenic hazardous substance that has to be managed with extreme caution. Caustic injury tends to be severe when the pH is above 11.5 [7]. In light of its chemical properties, the strong alkali produced from their external currents can cause serious colliquative necrosis and lipid saponification of the membrane when it reacts with the mucosa of the gastrointestinal tract. An oral toxicity study carried out in rats has shown that nickel hydroxide is associated with liver and intestine discoloration and increased mortality rate [8]. Nonetheless, there is no literature on the complications of the ingestion of NiMH batteries in humans. In this case, erosive gastritis was evident in <3 hours post-onset. It is therefore hypothesized that the ingestion of NiMH batteries can cause serious burns of gastrointestinal tract in a relatively short period of time. However, considering the fact that the patient had previous episodes of battery ingestion within 1 month, we could argue that the extensive mucosal burns found on gastroscopy were precipitated by previous battery ingestion. Therefore, further in-depth research is warranted to evaluate the potential harm of NiMH battery to human.

To our knowledge, this is the first described case of chemical burns of the stomach post-ingestion of NiMH battery in humans. Erosive gastritis can happen fairly rapidly, and gastroscopic retrieval should be considered if there is minimal advancement along the gastrointestinal tract. Lastly, close observation by carers is warranted to prevent the ingestion of batteries in children.
